# Radiation exposure in the endovascular therapy of cranial and spinal dural arteriovenous fistula in the last decade: a retrospective, single-center observational study

**DOI:** 10.1007/s00234-021-02816-6

**Published:** 2021-09-27

**Authors:** Marcel Opitz, Sebastian Zensen, Denise Bos, Yan Li, Hanna Styczen, Axel Wetter, Nika Guberina, Ramazan Jabbarli, Ulrich Sure, Michael Forsting, Isabel Wanke, Cornelius Deuschl

**Affiliations:** 1 Institute of Diagnostic and Interventional Radiology and Neuroradiology, Faculty of Medicine University Hospital Essen, University Hospital Essen, Hufelandstrasse 55, 45147 Essen, Germany; 2 Department of Diagnostic and Interventional Radiology, Neuroradiology, Asklepios Klinikum Harburg, Hamburg, Germany; 3 Department of Radiotion Therapy, University Hospital Essen, West German Cancer Center, Essen, Germany; 4 Department of Neurosurgery, University Hospital Essen, Essen, Germany; 5 Department of Neuroradiology, Clinic Hirslanden, Zurich, Switzerland

**Keywords:** Radiation exposure, Arteriovenous fistula, Embolization, Interventional neuroradiology

## Abstract

**Purpose:**

This study aims to determine local diagnostic reference levels (DRLs) in the endovascular therapy (EVT) of patients with cranial and spinal dural arteriovenous fistula (dAVF).

**Methods:**

In a retrospective study design, DRLs and achievable dose (AD) were assessed for all patients with cranial and spinal dAVF undergoing EVT (I) or diagnostic angiography (II). All procedures were performed at the flat-panel angiography-system Allura Xper (Philips Healthcare). Interventional procedures were differentiated according to the region of fistula and the type of procedure.

**Results:**

In total, 264 neurointerventional procedures of 131 patients with dAVF (94 cranial, 37 spinal) were executed between 02/2010 and 12/2020. The following DRLs, AD, and mean values could be determined: for cranial dAVF (I) DRL 507.33 Gy cm^2^, AD 369.79 Gy cm^2^, mean 396.51 Gy cm^2^; (II) DRL 256.65 Gy cm^2^, AD 214.19 Gy cm^2^, mean 211.80 Gy cm^2^; for spinal dAVF (I) DRL 482.72 Gy cm^2^, AD 275.98 Gy cm^2^, mean 347.12 Gy cm^2^; (II) DRL 396.39 Gy cm^2^, AD 210.57 Gy cm^2^, mean 299.55 Gy cm^2^. Dose levels of EVT were significantly higher compared to diagnostic angiographies (*p* < 0.001). No statistical difference in dose levels regarding the localization of dAVF was found.

**Conclusion:**

Our results could be used for establishing DRLs in the EVT of cranial and spinal dAVF. Because radiation exposure to comparably complex interventions such as AVM embolization is similar, it may be useful to determine general DRLs for both entities together.

## Introduction

Cranial dural arteriovenous fistulas (dAVFs) represent 10–15% of all intracranial vascular malformations with arteriovenous shunting and belong to the most frequently acquired vascular lesions of the central nervous system [1, 2]. The indication for treatment depends on the morphology of the cranial dAVF, the resulting probability of bleeding, and clinical presentation. High-grade fistulas type 2b-5 with cortical reflux classified by Cognard/Merland have a significantly higher risk of intracranial hemorrhage [3, 4]. In low-grade fistulas, type 1-2a by Cognard/Merland, a therapy refractory pulse-synchronous tinnitus is a typical treatment indication [5, 6]. Over the past decade, endovascular therapy (EVT) of patients with cranial dAVF evolved as the first-line treatment with high occlusion rates, low risk profile, and very low recurrence rates [7–9]. However, microsurgery, stereotactic radiosurgery, or combined therapy approaches remain as alternative treatment options.

Spinal dAVF represents the most common subset of spinal vascular malformations, accounting for approximately 70%. Nevertheless, it is a rare, probably underdiagnosed pathology with an incidence of only 5–10 new cases per million inhabitants per year [10]. In contrast to cranial dAVF, hemorrhage in spinal dAVF is very rare [11]. Nevertheless, a causal therapy is required in all patients because only the occlusion of the fistulous point will prevent progressive myelopathy caused by venous hypertension [12, 13]. Microneurosurgical occlusion of the fistula was the method of choice for many years, but more recently, endovascular techniques have augmented the therapeutic spectrum. Up to date, both endovascular and surgical treatment have been proven to be safe and effective [14, 15].

The role of DRLs in interventional neuroradiology has significantly increased over the last years as the guidelines for radiation protection have been updated recently [16, 17]. These minimally invasive fluoroscopically guided procedures are a highly effective treatment option for various neurovascular conditions. However, because of the complexity of the pathologies, some procedures may comprise high radiation exposure to patients and staff members [18, 19], leading to an increased potential deterministic and stochastic risk of developing radiation-induced cancer [20]. In order to raise dose awareness and in the long term optimize the modification of equipment, technique, and imaging parameters, several professional and regulatory organizations, such as the International Commission on Radiological Protection (ICRP), are proclaiming the necessity for diagnostic reference levels (DRLs), especially in interventional neuroradiology [21–23].

Data on radiation exposure of EVT in patients with cranial and spinal dAVF remain scarce. Hence, the goal of this study was to establish local DRLs at our department utilizing contemporary digital equipment.

## Methods

### Patient cohort

This retrospective study was approved by the ethical committee of our institution (20–9758-BO) and is conducted in accordance with the principles of the Declaration of Helsinki. All procedures were performed after written informed consent. The internal database was searched with an in-house-developed software for all consecutive diagnostic angiographies and endovascular treatments of cranial and spinal dAVF in the period between February 2010 to December 2020 (Table 1). All cranial dAVF were classified by Cognard/Merland type 1–5 (Table 2) [4].

**Table 1:** Demographic data and classification of dAVF

Parameter	Number (%)
Number of patients	131 (100%)
Cranial dAVF	94 (72%)
Male/female	60 (64%)/34 (36%)
Age (mean, range)	57, 23–83
Total number of EVT sessions	111
EVT with Onyx/EASYX	109 (98%)/2 (2%)
Frustrated EVT	5 (4%)
Additional platinum coils	24 (22%)
Additional ballon protection	29 (26%)
Additional surgery	6 (6%)
Spinal dAVF	37 (28%)
Male/female	26 (70%)/11 (30%)
Age (mean, range)	70, 30–79
Total number of EVT sessions/number of patients	24/ 22 (59%)
EVT with Glubran/Onyx	22 (92%)/2 (8%)
Frustrated EVT	3 (12%)
Additional platinum coils	2 (8%)
Additional surgery	4 (17%)
Exclusively surgery	15 (41%)

*dAVF* dural arteriovenous fistula, *EVT* endovascular therapy

**Table 2:** Classification of the 94 patients with cranial dural arteriovenous fistula (dAVF) according to Merland-Cognard Classification

Fistula type	Drainage pattern	*n* (%)
1	Drainage into dural venous sinus, antegrade flow	11 (11.7)
2a	Drainage into dural venous sinus, retrograde flow	24 (25.5)
2b	Drainage into dural venous sinus, antegrade flow, and cortical venous reflux	1 (1.0)
2a + b	Drainage into dural venous sinus, retrograde flow, and cortical venous reflux	14 (14.8)
3	Cortical venous reflux, no venous ectasia	8 (8.5)
4	Cortical venous reflux, venous ectasia	35 (37.2)
5	Drainage into spinal veins	1 (1.0)

### Procedure

All patients of this study cohort underwent diagnostic digital subtraction angiography (DSA) in house or external prior to EVT. DSA was performed to confirm the suspected diagnosis and classify the fistula for further tailoring the appropriate treatment. The decision to perform endovascular intervention was based on a case-by-case evaluation in an interdisciplinary decision-making process between neurosurgeons and interventional neuroradiologists. In the case of primary surgery in patients with spinal dAVF, DSA was performed subsequently for control purposes. All EVTs were performed under general anesthesia.

The standard EVT procedure of cranial dAVFs at our department is described in detail by Moenninghoff et al. [24]. A transfemoral access was gained via a 6-F sheath, and fluoroscopic guided superselective catheterization was performed to reach a wedge position with the microcatheter tip close to the fistula point. Almost all dAVFs were treated as the primary treatment by ethylene vinyl alcohol (EVOH) copolymer (Onyx®, Medtronic, Inc., Irvine, USA); only two patients received Easyx (Antia Therapeutics AG, Berne, Switzerland) alternatively. Superselective transarterial embolization technique and a detailed description of the EVOH liquid embolic system are stated in a precursor study of our department [9]. In some cases, an additional coil embolization or balloon protection of the venous sinus via venous transfemoral access was performed.

The standard EVT procedure of spinal dAVFs at our department is described in detail by Özkan et al. [25]. A transfemoral arterial approach was obtained, and under fluoroscopy, a guiding catheter was placed in the segmental artery. A microcatheter was introduced coaxially through the feeding pedicle and advanced into the distal aspect of a feeding artery close to the fistula in the ideal case in wedge-position so that a liquid embolic agent could be pushed up to the proximal venous side. In 20 patients, a mixture of Glubran® (cyanoacrylate glue, GEM s.r.l., Italy) and iodized oil (Lipiodol®, Guerbet, Aulnay-sous-Bois, France) in case-dependently variable concentrations (ranging from 1:3 up to 1:5) for appropriate flow characteristics and in two patients Onyx was injected. Ideally, a continuous injection embolized the feeding pedicle, including the terminal feeders up to the fistulous point, as well as the beginning of the early draining vein. A final spinal angiogram of the initially feeding segmental artery and of the adjacent and contralateral segmental arteries was performed.

The intervention was considered successful if embolization of the dAVF was possible. In a few cases, an endovascular embolization attempt was made, but failed and was considered frustrating.

### Biplanar angiography system

All procedures were performed at the Allura Xper FD20/10 system (Philips Healthcare, Eindhoven, The Netherlands) by an experienced team of neuroradiologists. As we are a university hospital, young neuroradiologists were regularly involved in the interventions in addition to a neuroradiologist with many years of angiography experience. The X-ray unit is equipped with automatic control dose rate system. The frame rate frequently used at pulsed fluoroscopy mode was 1 pulse/s. The focus-to-skin distance varied from 60 to 70 cm. The Allura Xper system has one detector 20-inch with a maximum field of view (FOV) of 48 cm and one 10-inch detector with a max. FOV of 25 cm. The deposited protocol for the treatment of dAVF was set at a characteristic tube voltage of 80 kV. An anti-scatter grid and an aluminum filter with 1-mm thickness were used. To test system performance and stability over time, periodic quality controls were performed during maintenance visits.

### Dose calculation

Radiation exposure for diagnostic DSA and EVT was determined in terms of dose area product (DAP). To achieve dose optimization in the clinical routine, DRLs are a globally accepted parameter for dose monitoring, in the interventional setting typically defined in terms of the DAP. DRLs represent the 75th percentile of a dose distribution of a specific radiological procedure and may indicate whether the radiation dose lies within the normal range of a dose distribution at radiological departments [26, 27]. The achievable dose (AD) is another important parameter for dose optimization representing the median of a dose distribution [28].

### Statistical analysis

The interventions were analyzed according to the type of procedure and the type of fistula. The mean, median, and 75th percentile of the DAP, as well as the mean fluoroscopy time, were calculated. A *p*-value lower than 0.05 was considered as statistically significant. Statistical analysis was performed with the Statistical Package for Social Sciences v. 27.0. (SPSS Inc., New York, USA).

## Results

### Patient cohort

Between February 2010 and December 2020, 264 consecutive neurointerventional procedures in 131 patients with dAVF (94 cranial, 37 spinal) were performed in our department. The median age of patients with cranial and spinal dAVF was 60 years (range 23–87 years) and 70 years (range 30–79 years), respectively. The gender distribution in both cohorts was in favor of the male gender (cranial dAVF 60/94; spinal dAVF 26/37). Out of 94 patients with cranial dAVF, 111 EVTs were performed using Onyx/EASYX (109/ 2). A successful embolization was achieved in 106 out of 111 interventions (95.5%). Out of 37 patients with spinal dAVF, 22 received EVT with Glubran/ Onyx and 15 patients underwent primary surgery. Embolization was in 21/24 (87.5%) procedures successful (Table 1).

### Radiation exposure and DRLs

The following DRLs, AD, and mean values could be determined for all patients with dAVF undergoing EVT (I) or diagnostic cerebral angiography (II): for cranial dAVF (I) DRL 507.33 Gy cm^2^, AD 369.79 Gy cm^2^, mean 396.51 Gy cm^2^; (II) DRL 256.65 Gy cm^2^, AD 214.19 Gy cm^2^, mean 211.80 Gy cm^2^; for spinal dAVF (I) DRL 482.72 Gy cm^2^, AD 275.98 Gy cm^2^, mean 347.12 Gy cm^2^; (II) DRL 396.39 Gy cm^2^, AD 210.57 Gy cm^2^, mean 299.55 Gy cm^2^ (Table 3).

**Table 3:** Distribution of total DAP as a function of procedure type and dural arteriovenous fistula site

**Location of dAVF**	**Type of procedure**		**Total DAP (Gy cm**^**2**^**)**				**FT**
		**n**	**25th percentile**	**Median**	**75th percentile**	**Mean**	**Mean**
Cranial	DCA	71	129.57	214.19	256.65	211.80	17 min 18 s
	EVT	111	264.65	369.79	507.33	396.51	58 min 57 s
Spinal	DCA	58	125.57	210.57	396.39	299.55	25 min 33 s
	EVT	24	169.37	275.98	482.72	347.12	35 min 45 s

*DCA* diagnostic cerebral angiography, *DAP* dose area product in gray per square centimeter, *dAVF* dural arteriovenous fistula, *EVT* endovascular treatment, *FT* fluoroscopic time in minutes, *n* number of studies

### Comparison of radiation exposure regarding the type of fistula and procedure

**Fig. 1:**
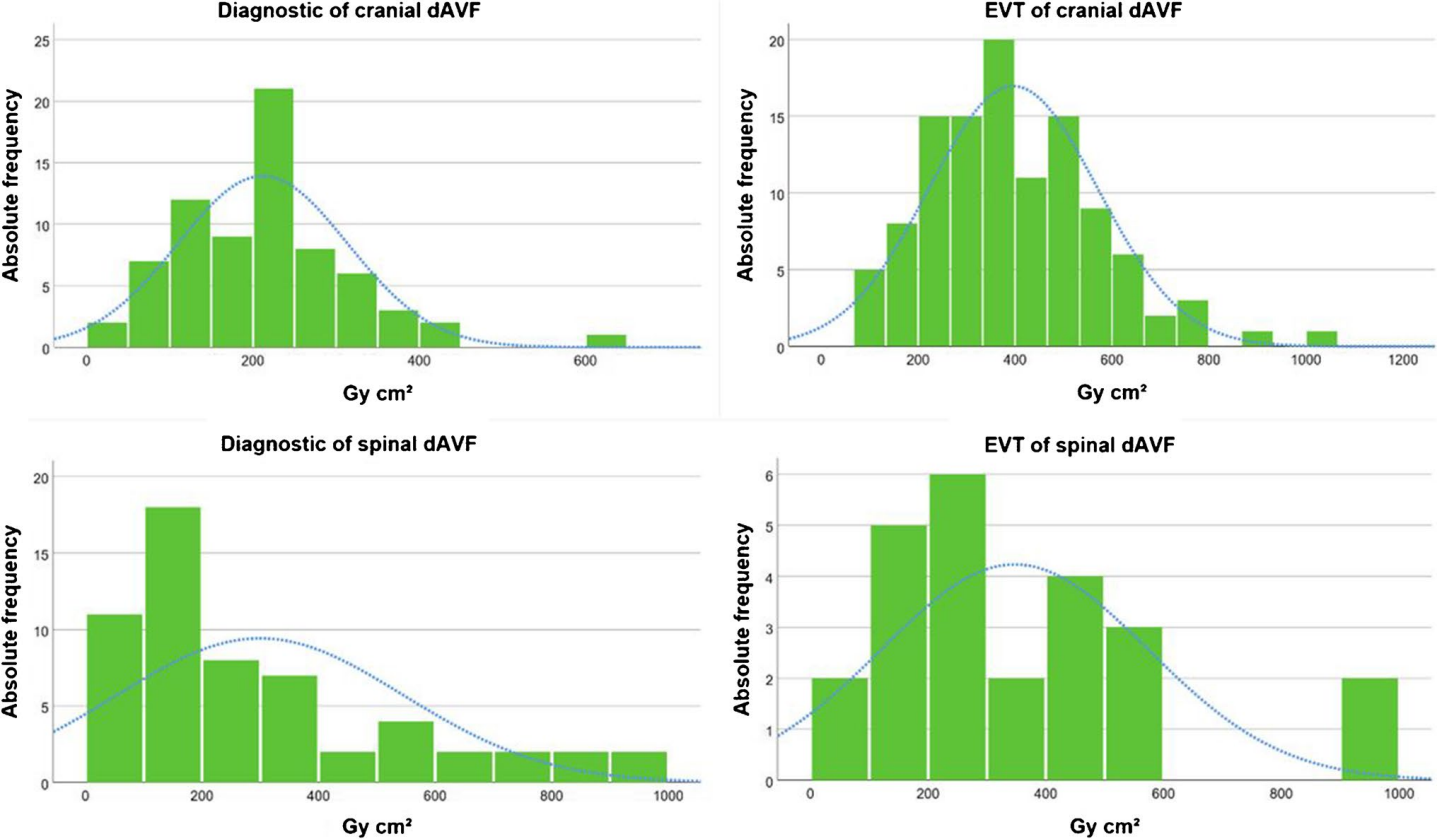
Histogram of dose area product (Gy cm^2^) for diagnostic DSA and endovascular therapy (EVT) of cranial and spinal dAVF; blue curve highlighting distribution graph

**Fig. 2:**
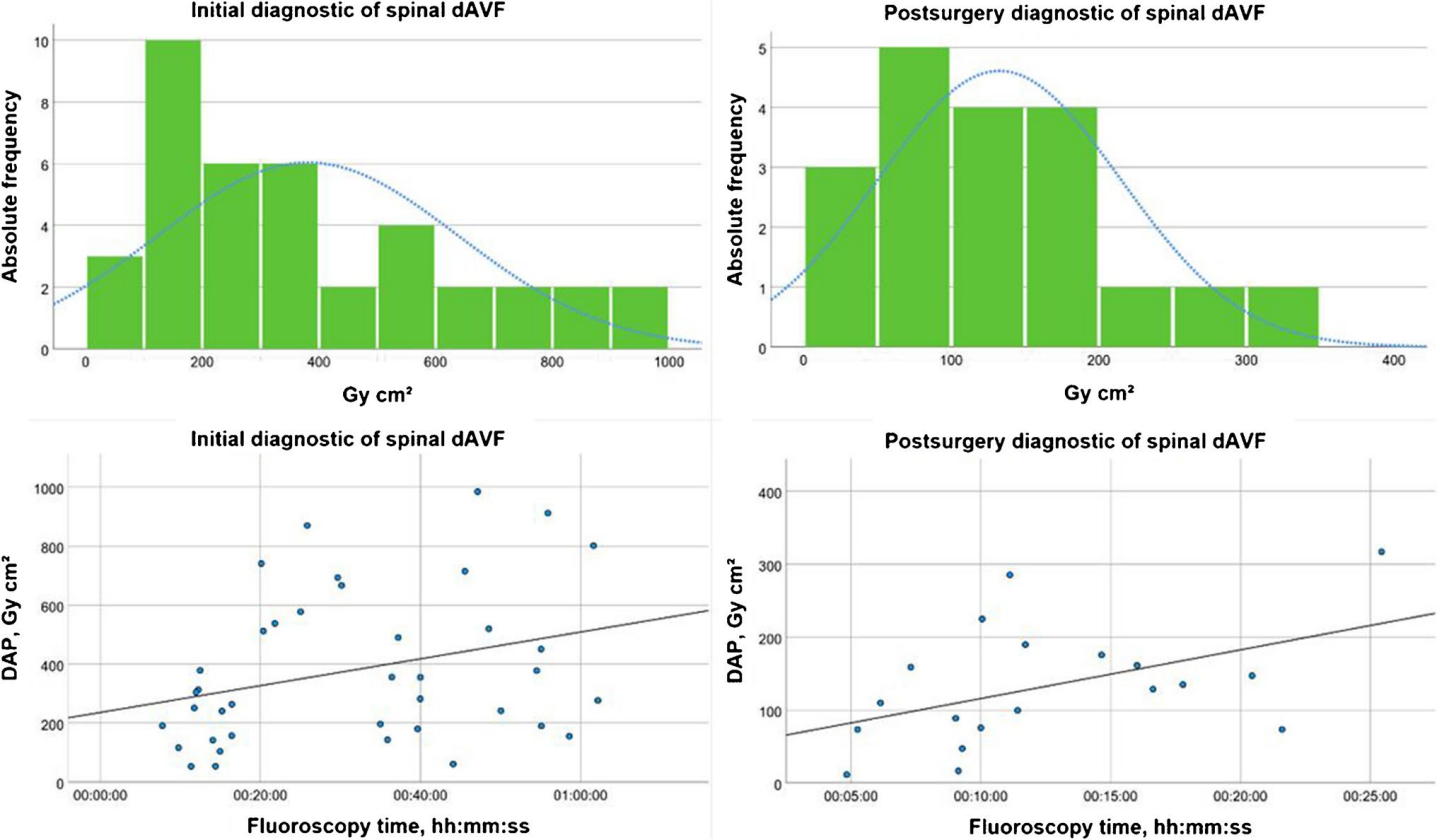
Histogram of dose area product (Gy cm^2^) and scatter plot with adjustment line between dose area product (DAP) and fluoroscopy time for initial and postsurgery diagnostic DSA in patients with spinal dAVF

The Kruskal–Wallis test with Dunn-Bonferroni post hoc test revealed for both cranial and spinal dAVF a significant dose difference regarding the type of procedure (*p* < 0.001) (Fig. 1). No statistical dose difference was found between the different region of fistula (cranial vs. spinal) according to DSA (*p* = 0.380) and EVT (*p* = 0.472). As normal distribution was fulfilled and the Levene´s test confirmed the equality of variance between the subgroups Cognard 4 and 2a in patients with cranial dAVF (*p* = 0.685), the *t*-test was applied. No significant differences of DAP between the two subgroups (*p* = 0.548) were observed. The one-way ANOVA confirmed a significant dose difference between initial DSA and postsurgery DSA in patients with spinal dAVF (*p* < 0.001) (Fig. 2). Excluding the frustrated therapy sessions, no significant difference of DAP was observed for cranial dAVF (*p* = 0.932) or spinal dAVF (*p* = 0.076). Likewise, no significant difference of DAP was found for cranial dAVF (*p* = 0.151) or spinal dAVF (*p* = 0.873) by excluding all the patients who underwent more than one therapy session.

**Fig. 3:**
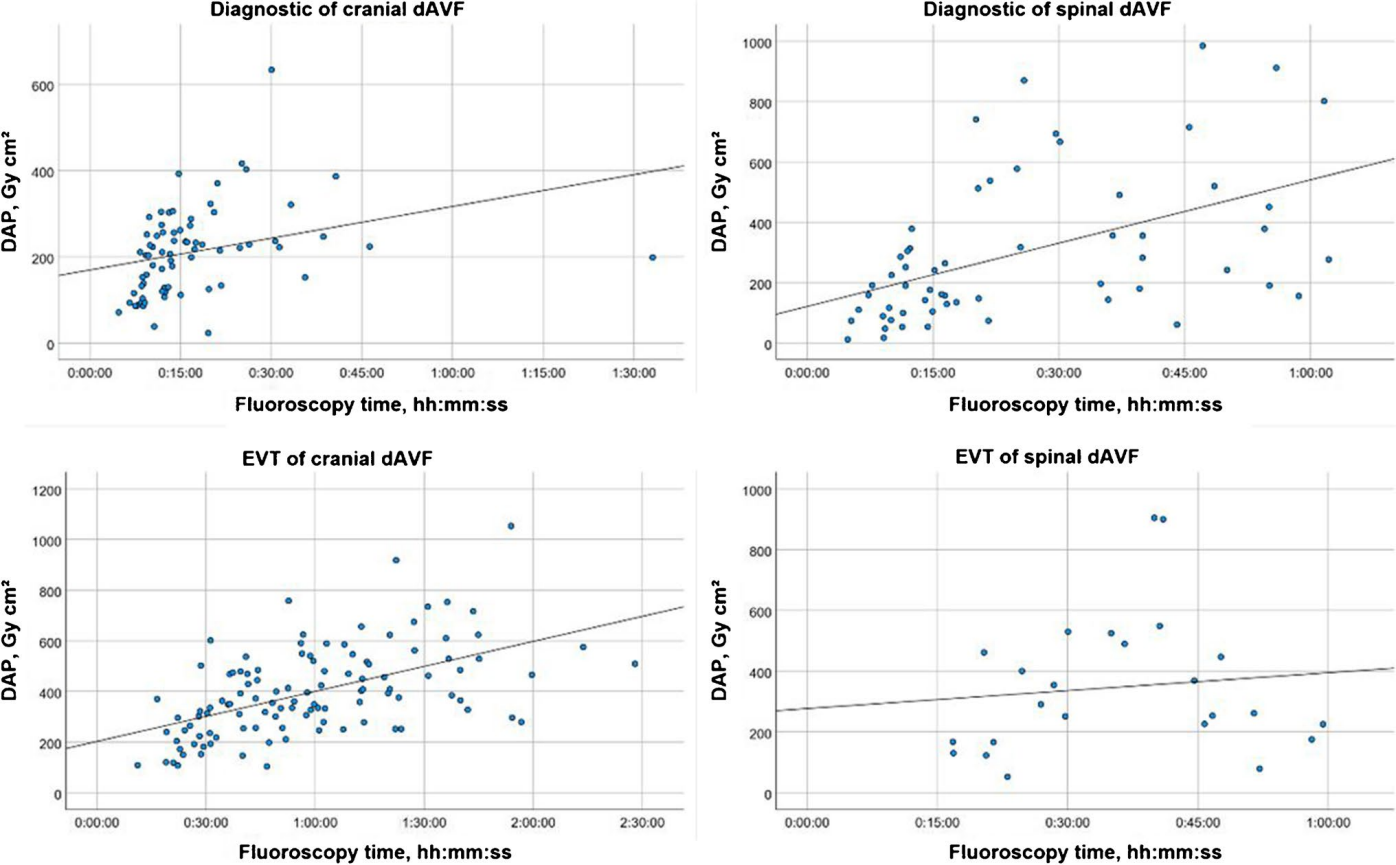
Scatter plot with adjustment line between dose area product (DAP) and fluoroscopy time for diagnostic DSA and endovascular therapy (EVT) of cranial and spinal dAVF

The mean fluoroscopy times (FT) are listed in Table 3. Spearman correlations were run to assess the linear relationship between DAP and FT for DSA and EVT (Fig. 3).

## Discussion

Our study analyzes radiation exposure of fluoroscopy-guided angiographies of patients with dAVF and reveals useful dose data differentiated by the type of fistula, anatomical region of fistula, and procedure. The results may be used as a benchmark for the national radiation protection authorities to implement DRLs in the EVT of cranial and spinal dAVF as proposed by the European Directive 2013/59/Euratom [23].

With regard to interventional neuroradiology, until now, the German Federal Office for Radiation Protection only published DRLs for thrombus aspiration (DRL 180.0 Gy cm^2^) and aneurysm coiling (DRL 250.0 Gy cm^2^) [29]. For dAVF embolization, only few authors addressed the issue of radiation exposure at all. Forbig et al. provided detailed dosimetry data for the endovascular treatment of intracranial lateral dAVF differentiated by the Cognard grade and endovascular technique [30]. The proposed DRLs are slightly lower compared to our study (DRL 414.0 Gy cm^2^). This is probably related to the strict selection criteria. In our study, we also excluded other intracranial fistulae, such as CCF. However, in their study, anterior crainal fossa dAVF were also excluded. Other studies neither yielded information concerning the dedicated type of dAVF nor the applied endovascular approach [31, 32].

The local DRLs determined in our study (dAVF cranial 507.3 Gy cm^2^ and spinal 482.7 Gy cm^2^) are higher, but in the range of the published data of other studies describing the radiation exposure of AVM embolization only, e.g., Miller et al. (479,2 Gy cm^2^ cranial AVM, 476,3 Gy cm^2^ spinal AVM) and Kien et al. (440 Gy cm^2^ cranial AVM) [33, 34]. Since EVT of both cerebrovascular malformations are complex neurointerventional procedures with a similar therapeutic approach, it may not surprise that our DRLs are in a similar range.

As shown in previous studies, the amount of radiation for interventional procedures is much more affected by procedure complexity than by patient size and weight [35]. Therefore, DRLs for interventional procedures should be ideally established according to the type and complexity level of the procedure. Although the EVT of cranial and spinal dAVF is performed in different anatomical regions, the therapeutic approach is similar, and both are complex interventional procedures. This may explain why we could not find any significant dose differences in the EVT of both fistula types in our cohort. Since our DRLs are in a similar range to the published local DRLs for embolization of AVM, it is worth discussing whether DRLs should be defined under the umbrella term EVT of cerebrovascular malformations.

The failure of a dAVF embolization only becomes apparent in the course of the procedure and frustrated fluoroscopy-guided therapy sessions can involve a similarly high radiation dose as successful procedures. This may explain why in our study no significant difference of DAP was found by excluding the frustrated therapy sessions.

Excluding all the patients who underwent more than one therapy session did not affect the total DAP in the EVT of cranial and spinal dAVF. However, most patients received only one therapy session, so conclusions about dose differences are obsolete.

As shown in Figs. 2 and 3, there is a linear correlation between the DAP and the FT, but the FT is a poor predictor of dose to the patient, because it does not account for the effects of image acquisition modes. To estimate stochastic risks of radiation exposure, the effective dose is a more straightforward value [36]. However, to compare radiation exposure of different devices at different sites in the clinical routine, DRLs are a practical and a globally accepted parameter for dose monitoring.

Patients with spinal dAVF received an initial DSA and in some cases also a postoperative control DSA to ascertain successful elimination of the fistula. The initial DSAs are more time-consuming in clinical routine and usually require more sequences due to the complexity of the disease. For this reason, it is not surprising that a significantly higher dose was determined for the initial DSA compared to the post-surgery DSA (Fig. 2).

Cranial dAVF type 2a are confined to sinus, and consequently, the fistula point is easier to reach than in type 4 fistula, which drains directly into cortical veins. However, with regard to radiation exposure, no significant difference was found in our study. Consequently, it does not seem to be useful to distinguish DRLs between the different fistula types according to Cognard.

It is striking that the gender distribution in our study clearly favors the male gender. This finding is consistent with studies that have shown that men are more prone to cranial and spinal dAVF than women [24, 37].

The most important limitation of our study is the retrospective and unicenter design with different cohort sizes. Our determined dose levels may differ from those of other sites and angiography devices. Therefore, the examination of radiation exposure at different sites and devices are the next necessary steps for the determination of national and European DRLs. An experienced team of neuroradiologists performed all procedures, but on a university hospital, young neuroradiologists are also trained. In terms of radiation dose, our results therefore may indicate higher doses than can possibly be achieved.

Strengths of our study include the large number of datasets collected on the same biplanar angiography system enabling specific dose assessment. To determine local DRLs for a single center, it is recommend by Vano et al. to collect the radiation data of more than 50 examinations within the same type of procedure because of the high individual variability of interventional procedures [38]. In this study, the number of procedures was greater than 50 for the EVT of cranial dAVFs. In rare interventional procedures, DRLs may also be determined for more than 20 examinations, as in our study for the EVT of spinal dAVF.

## Conclusion

Increasing regulatory requirements necessitate dose monitoring of patients and staff members, and justification of aberrant exposures. This is the first comprehensive data acquisition of radiation exposure during dAVF therapy in a neuroradiology referral centre, which explicitly distinguishes between EVT of cranial and spinal dAVF. Although EVT was performed in two different anatomical regions, no significant dose difference was found between the two entities. Because radiation exposure to comparably complex interventions such as AVM embolization is similar, it may be useful to determine general DRLs for both entities together.

## Abbreviations

AD: Achievable dose
AVM: Arteriovenous malformation
CCF: Carotid-cavernous fistula
DAP: Dose area product
dAVF: Dural arteriovenous fistula
DRLs: Diagnostic reference levels
EVT: Endovascular therapy
FOV: Field of view
FT: Fluoroscopy time
ICRP: International Commission on Radiological Protection
